# Nursing students' attitudes towards sexuality before training in sexual and reproductive health

**DOI:** 10.1002/nop2.1959

**Published:** 2023-07-29

**Authors:** Sergio Martínez‐Vázquez, Antonio Hernández‐Martínez, Rocío Adriana Peinado‐Molina, Juan Miguel Martínez‐Galiano

**Affiliations:** ^1^ Department of Nursing of the University of Jaen Jaen Spain; ^2^ Department of Nursing, Faculty of Nursing of Ciudad Real University of Castilla‐La Mancha Ciudad Real Spain; ^3^ Consortium for Biomedical Research in Epidemiology and Public Health (CIBERESP) Madrid Spain

**Keywords:** attitudes, human and sexual rights, nursing students, sexual health, training

## Abstract

**Aim:**

To know the attitudes towards sexuality of nursing students and those sociodemographic and cultural factors that can influence it.

**Design:**

An observational study was carried out on Nursing degree students. One hundred and eighteen nursing degree students who were going to take the sexual and reproductive health nursing course.

**Methods:**

A self‐administered online questionnaire. This questionnaire had several parts specifically designed to collect sociodemographic variables. In order to determine the attitudes towards sexuality, the questionnaire based on the ATSS (Attitudes Towards Sexuality Scale). The Double Standard Scale (DSS) was also used. This scale evaluates double standards within the area of sexuality. The Rape Supportive Attitude Scale (RSAS), was included to determine beliefs about rape, rapists and their victims.

**Results:**

The ATSS shows a statistically significant association with religious beliefs (*p* = 0.005), with mean scores of 113.84 (SD = 9.81) for non‐believers (no religion), 108.36 (SD = 15.68) for non‐practicing believers and 102.32 (SD = 17.87) for believers (those who practice their faith). The mean score in the DSS shows a statistically significant association with alcohol consumption (*p* = 0.001). The mean score on the RSAS is statistically significantly associated with the place of residence (*p* = 0.050), the means were 44.32 (SD = 9.26) for those who lived in the city, 34.94 (SD = 19.21) if the place of residence was between 10,000 and 5000 inhabitants and 32.54 (SD = 15.01) if the family home was in a town with less than 5000 inhabitants.

**Conclusion:**

Religious beliefs reduce liberalism and positive attitudes towards sexuality, whereas sporadic alcohol consumption increases them.

**Patient or Public Contribution:**

No patient or public contribution.

## 1 INTRODUCTION

Sexuality is an inherent aspect of being human and includes gender identity, sexual orientation, affectivity, affective relationships, pleasure and reproduction (Foucault, 2019). The World Health Organization (WHO) defines sexual health as: ‘a state of physical, mental, and social well‐being in relation to sexuality. It requires a positive and respectful approach to sexuality and sexual relations, as well as the possibility of having pleasurable and safe sexual experiences, free from all coercion, discrimination, and violence’ (World Health Organization, 2017). This definition emphasizes the fact that sexual health is more than the absence of disease, it is a complex construct formed by biological, psychological and sociocultural dimensions and is influenced by economic and historical factors, among others (Foucault, 2019).

The study of sexuality should be addressed, normalized and integrated into the academic curriculum without focusing solely on disease prevention (Gómez‐Zapiaín et al., 2016). This is to include the gender variants that have emerged in recent decades, sexual attitudes and behaviours, respect for human and sexual rights and attachment (Gómez‐Zapiaín et al., 2016; Pavelová et al., 2021). It should also include sexual diversity, tolerance and understanding (Etchezahar et al., 2016; Gómez‐Zapiaín et al., 2016; Moral & Valle, 2012; Páez et al., 2015; Pavelová et al., 2021; Sierra et al., 2007).

The training of future health professionals should include this profile and the proper management of contraceptive methods (Fisher & Hall, 1988; Gómez‐Zapiaín et al., 2016). As the nursing degree progresses, a more positive attitude towards sexuality is shown (Gorrotxategi et al., 2020). Although some authors affirm a lack of sufficiently comprehensive evaluation of sexuality in the nursing studies curriculum, highlighting, in particular, the lack of evaluation of the competencies of the students and even of the professors (Dawson et al., 2016; de Vries et al., 2020). Sexual health educational needs of university students in health sciences should not be overlooked or may even include their parents (Uğurlu & Karahan, 2022).

The involvement of students and the attitude they have during the learning process has a great impact on their training, especially in terms of clinical skills (Edgecombe et al., 2013). The role of the teacher continues to be a fundamental pillar in the education of the nursing student, even in contexts where education has had to adapt to other formats (McDonald et al., 2018; Sozeri Ozturk et al., 2022). Teachers can stimulate their attitude, enhancing their learning capacity (Arreciado Marañón & Isla Pera, 2015). The teacher who knows their students' attitudes well can work with them (Murdoch et al., 2017; Puplampu, 2017), and this knowledge is fundamental in the planning and development of teaching.

In the current educational context, it is necessary to know and identify the previous skills of the students, and their attitudes towards sexuality before starting training in this subject, to understand which areas need to be strengthened throughout the training in the subject, along with better understanding what needs must be met in the acquisition of concepts and skills (Gómez‐Zapiaín et al., 2016; Pavelová et al., 2021).

For all these reasons, the main objective is to know the attitudes towards sexuality of nursing students and those sociodemographic and cultural factors that can influence it.

## 2 MATERIALS AND METHODS

### 2.1 Design and sample selection

An observational study was carried out on Nursing degree students at the University of Jaén who were going to take the sexual and reproductive health nursing course in the 2022–2023 academic year. Inclusion criteria were established as being 18 years old or older and being enrolled in the sexual and reproductive health nursing course of the Nursing Degree at the University of Jaén. In addition, not having received prior sexual and reproductive health training was established as an exclusion criterion. Those students who did not want to participate were excluded. For the sample calculation, the following criteria were used to estimate a population mean where a standard deviation of 12.61 was considered for the ATTS scale (Alonso‐Martínez et al., 2022), with an infinite population, a precision of 3 points, and a replacement percentage of 10%, estimating a minimum of 76 students. Given the possible loss during the study and to improve statistical power, it was decided to include all the students who wanted to participate.

### 2.2 Information source and study variables

The data were collected in 2022. A self‐administered online questionnaire was used that included an informed consent that the students had to accept before filling out the questionnaire. This questionnaire had several parts specifically designed to collect sociodemographic variables. In order to determine the attitudes towards sexuality, the questionnaire based on the ATSS (Attitudes Towards Sexuality Scale) of 14 items, by Fisher and Hall (1988) was used, subsequently expanded to 28 items and validated in the Spanish context (Ruibal et al., 2005). The score varies from 28 to 140 points. Lower values indicate a lower degree of liberalism and more negative attitudes towards sexuality (Ruibal et al., 2005). The Double Standard Scale (DSS) was also used, which is a scale made up of 10 items, which are answered using a Likert‐type scale of scores from 1 to 5. This scale evaluates double standards within the area of sexuality. The scale, originally developed by Caron et al. (1993) has been recently validated and used in a Spanish context (Diéguez, 2003; Ruibal et al., 2005). The scores of the scale items vary from 10 to 50 points. Higher values indicate high adherence to the sexual double standard (Diéguez, 2003; Ruibal et al., 2005). A final scale, the Rape Supportive Attitude Scale (RSAS), was included to determine beliefs about rape, rapists and their victims. This scale uses a Likert‐type score from 1 to 5, where 5 means totally agree. This scale was designed by Lottes (1991, 1998) and later used in Spanish context (Sierra et al., 2007). All items are scored in the same direction. The higher the score (20–100), the more attitudes supportive of rape or insensitivity to the victim are supported by a respondent (Sierra et al., 2007). In this way, it is possible to know, in addition to the sexual attitudes of the students, macho attitudes and prejudices. All the scales used have been validated in similar population of the study's sample (Diéguez, 2003; Ruibal et al., 2005; Sierra et al., 2007).

The independent variables were sociodemographic, such as age, biological sex, gender identity, sexual identity, marital status, religiosity or place of family residence, among others. The main dependent variable used were attitude towards sexuality, double standards within the area of sexuality, macho attitudes, prejudices and beliefs about rape, rapists and victims. The questionnaire was provided to the students on the first day of class before the teaching of any content of the subject.

### 2.3 Statistical analysis

First, descriptive statistics were carried out using absolute and relative frequencies in the qualitative variables. For the bivariate analysis, the Student‐Fisher *t* test was used when the independent variable presented two categories, the analysis of variance test (ANOVA) for variables with more than two categories and linear regression when the independent variable was quantitative. The study of the relationship between the three scales was carried out using the Pearson correlation coefficient. All analyses were performed with SPSS.

### 2.4 Ethical considerations

The study received a favourable report from the Research Ethics Committee of the University of Jaen with reference number SEPT.22/4.PRY. Before starting the questionnaire, the students had to read an information sheet about the study and its objectives and check a box confirming their consent to participate in it; that is, they signed a digital informed consent elaborated specifically for the study and the way of collecting the information.

## 3 RESULTS

A total of 118 nursing degree students from the University of Jaén participated. All of them were second‐year students, and they began their official degree training in sexual and reproductive health that day. The mean age was 21.81 (SD = 6.01). Of the participants, 98.3% (116) declared they were cisgender, 44.1% (52) had a partner and 50% (59) were single. Regarding sexual identity, 78% (82) reported being heterosexual, 17.8% (21) bisexual and 2.5% (3) homosexual. A total of 75.4% (89) did not smoke, and 72% (85) consumed alcohol sporadically. A total of 73.7% (87) have their family home in a city, and 89.8% (106) did not suffer from any chronic disease. The rest of the information that characterizes the sample and their score in the different scales can be consulted in Table 1. [Correction added on 5 August 2023 after first online publication: ‘University of REDACTED’ has been updated to ‘University of Jaén’ under sections “Design and sample selection” and “RESULTS”]

**TABLE 1:** Sociodemographic characteristics of the sample.

Variables	Total, *N* (%)
Age	
Mean (SD)	21.85 (6.01)
Gender	
Cisgender	116 (98.3)
Non‐binary	2 (1.7)
Married status	
Single	59 (50.0)
Married	5 (4.2)
W/Partner	52 (44.1)
Divorced	1 (0.8)
Other	1 (0.8)
Spiritual beliefs (religiosity)	
No	51 (43.2)
Yes, non‐practicing	45 (38.1)
Yes, practicing	22 (18.6)
Sexual Identity	
Heterosexual	82 (78.0)
Homosexual	3 (2.5)
Bisexual	21 (17.8)
Pansexual	1 (0.8)
Antrosexual	1 (0.8)
Tobacco use	
Sporadically	18 (15.3)
No	89 (75.4)
Yes, frequently	11 (9.3)
Alcohol use	
Sporadically	85 (72.0)
No	32 (27.1)
Yes, frequently	1 (0.8)
Recreational drugs use	
Sporadically	2 (1.7)
No	116 (98.3)
Yes, frequently	0 (0.0)
Has child(ren)	
No	113 (95.8)
Yes	5 (4.2)
Place of residence (population)	
>10,000 inhabitants	87 (73.7)
5000–10,000 inhabitants	18 (15.3)
<5000 inhabitants	13 (11.0)
Chronic illness	
No	106 (89.8)
Yes	12 (10.2)

**FIGURE 1:**
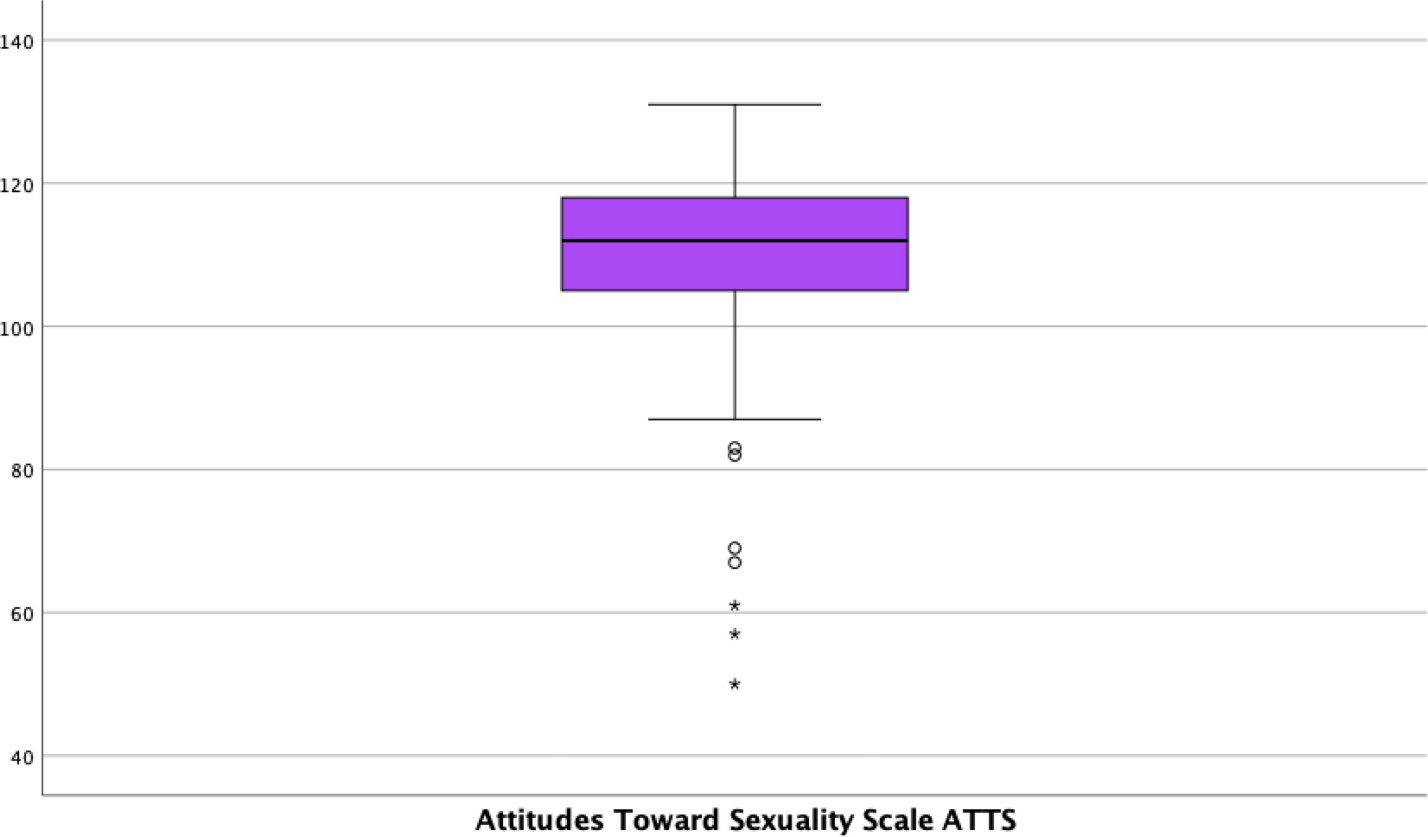
Boxplot of scoring. Attitudes Toward Sexuality Scale (ATSS).

Regarding attitudes towards sexuality, the mean score in the ATSS was 109.60 (SD = 14.47). The distribution of scores by items in the questionnaire can be consulted in Table S1, and the mean distribution of scores can be seen in Figure 1. The results of the ANOVA indicated that the mean score in the ATSS shows a statistically significant association with religious beliefs (*p* = 0.005), with mean scores of 113.84 (SD = 9.81) for non‐believers (no religion), 108.36 (SD = 15.68) for non‐practicing believers and 102.32 (SD = 17.87) for believers (those who practice their faith). Alcohol consumption (*p* = 0.018) also shows a statistically significant association with mean scores with 111.93 (SD = 10.48) for occasional consumption, 103.59 (SD = 20.95) for no consumption and 104.00 (SD = 0.0) for occasional consumption. frequent. The rest of the information from the bivariate analysis can be found in Table 2.

**TABLE 2:** ANOVA and the significance of comparing samples and their scores on the scales used.

Variables	ANOVA								
	ATSS, mean (SD)	*F*	Sig.	RSAS, mean (SD)	*F*	Sig.	DSS, mean (SD)	*F*	Sig.
Age									
Mean (SD)	109.60 (14.47)			30.35 (9.93)			43.54 (10.57)		
Gender									
Cisgender	109.53 (14.57)	0.147	0.702	30.42 (1.00)	0.387	0.535	43.47 (10.64)	0.360	0.550
Non‐binary	113.50 (7.78)			26.00 (5.66)			48.00 (1.41)		
Married status									
Single	108.85 (13.65)	0.909	0.461	31.32 (10.99)	0.758	0.555	44.14 (10.10)	0.204	0.936
Married	100.00 (23.60)			35.20 (7.66)			40.80 (10.33)		
W/Partner	111.06 (14.48)			28.89 (8.88)			43.25 (11.41)		
Divorced	115.00 (0.00)			27.00 (0.00)			43.00 (0.00)		
Other	121.00 (0.00)			28.00 (0.00)			38.00 (0.00)		
Spiritual beliefs (religiosity)									
No	113.84 (9.81)	5.546	**0.005**	29.88 (7.54)	0.104	0.902	43.49 (10.35)	0.024	0.976
Yes, non‐practicing	108.36 (15.68)			30.80 (11.26)			43.78 (10.95)		
Yes, practicing	102.32 (17.87)			30.50 (12.17)			43.18 (10.76)		
Sexual identity									
Heterosexual	108.63 (15.50)	0.646	0.631	30.82 (10.83)	0.316	0.867	43.63 (10.51)	0.746	0.562
Homosexual	113.00 (8.89)			30.67 (8.02)			33.67 (18.23)		
Bisexual	113.52 (9.78)			28.29 (5.58)			44.33 (10.04)		
Pansexual^a^	115.00 (0.00)			27.00 (0.00)			43.00 (0.00)		
Antrosexual^b^	101.00 (0.00)			33.00 (0.00)			49.00 (0.00)		
Tobacco use									
Sporadically	115.17 (10.08)	1.825	0.166	28.83 (5.01)	1.340	0.266	44.67 (8.94)	1.478	0.232
No	108.94 (14.78)			31.13 (11.03)			42.73 (11.35)		
Yes, frequently	105.82 (16.71)			26.45 (3.91)			48.27 (2.33)		
Alcohol use									
Sporadically	111.93 (10.48)	4.145	**0.018**	28.71 (5.47)	4.731	**0.011**	45.62 (8.20)	7.231	**0.001**
No	103.59 (20.95)			34.81 (16.23)			37.81 (13.87)		
Yes, frequently	104.00 (0.00)			27.00 (0.00)			50.00 (0.00)		
Recreational drugs use									
Sporadically	104.50 (13.44)	0.251	0.617	25.00 (1.41)	0.587	0.445	48.50 (0.71)	0.445	0.506
No	109.69 (14.52)			30.44 (10.00)			43.46 (10.64)		
Yes, frequently	0 (0.0)			0 (0.0)			0 (0.0)		
Has child(ren)									
No	109.96 (13.82)	1.605	0.208	29.88 (8.99)	6.271	**0.014**	43.81 (10.34)	1.775	0.185
Yes	101.60 (26.26)			41.00 (21.90)			37.40 (14.94)		
Place of residence (population)									
>10,000 inhabitants	111.00 (12.00)	1.593	0.208	29.07 (5.52)	3.067	**0.050**	44.32 (9.26)	1.809	0.168
5000–10,000 inhabitants	106.22 (20.46)			34.94 (18.21)			43.50 (13.01)		
<5000 inhabitants	104.92 (18.99)			32.54 (15.01)			38.38 (14.20)		
Chronic illness									
No	110.44 (12.69)	3.606	0.060	29.84 (7.87)	2.763	0.099	44.29 (9.50)	5.448	**0.021**
Yes	102.17 (24.95)			34.83 (20.85)			36.92 (16.53)		

^a^ Sexual attraction to some people, regardless of their biological sex or gender identity.

^b^ This concept encompasses those who experience their sexuality without knowing in which category to identify themselves and/or without feeling the need to classify themselves in any of them can identify with it.

Bold means statistically significant.

**FIGURE 2:**
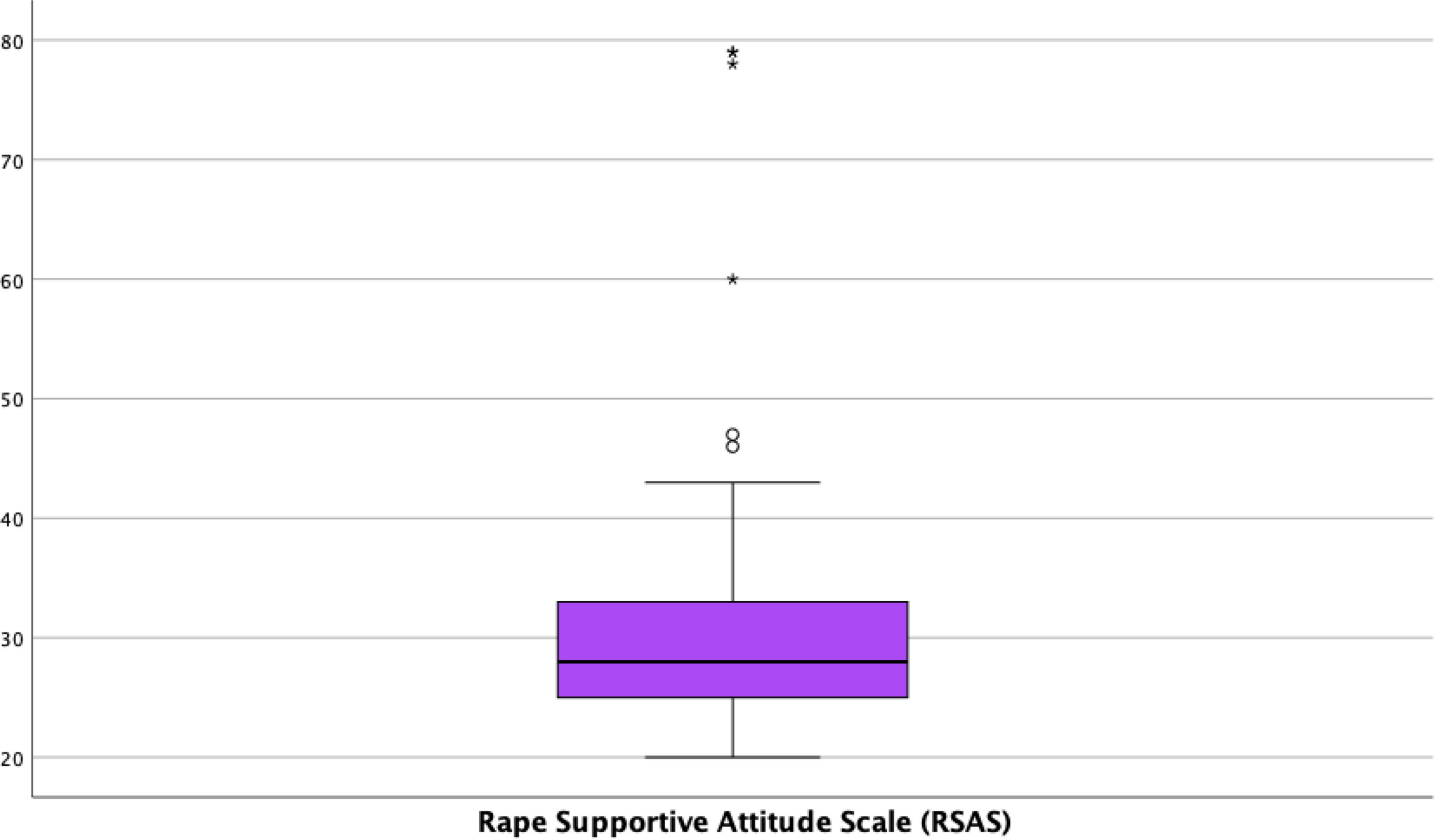
Boxplot of scoring. Rape Supportive Attitude Scale (RSAS).

For the study of perceptions of rape, rapists and their victims, a mean score on the RSAS of 43.54 (SD = 10.57) was found. The distribution of scores by items in the questionnaire can be found in Table S2, and the mean distribution of scores can be seen in Figure 2. The results of the ANOVA found that the mean score on the RSAS is statistically significantly associated with having children (*p* = 0.014), with means of 29.88 (SD = 8.99) for no‐children and 41.00 (SD = 21.90) for those who had children. An association was also found with alcohol consumption (*p* = 0.011), with means of 28.71 (SD = 5.47) for sporadic consumption, 34.81 (SD = 16.23) for no consumption and 27.00 (SD = 0.0) for habitual consumption. Regarding the place of residence (*p* = 0.050), the means were 44.32 (SD = 9.26) for those who lived in the city, 34.94 (SD = 19.21) if the place of residence was between 10,000 and 5000 inhabitants and 32.54 (SD = 15.01) if the family home was in a town with less than 5000 inhabitants.

**FIGURE 3:**
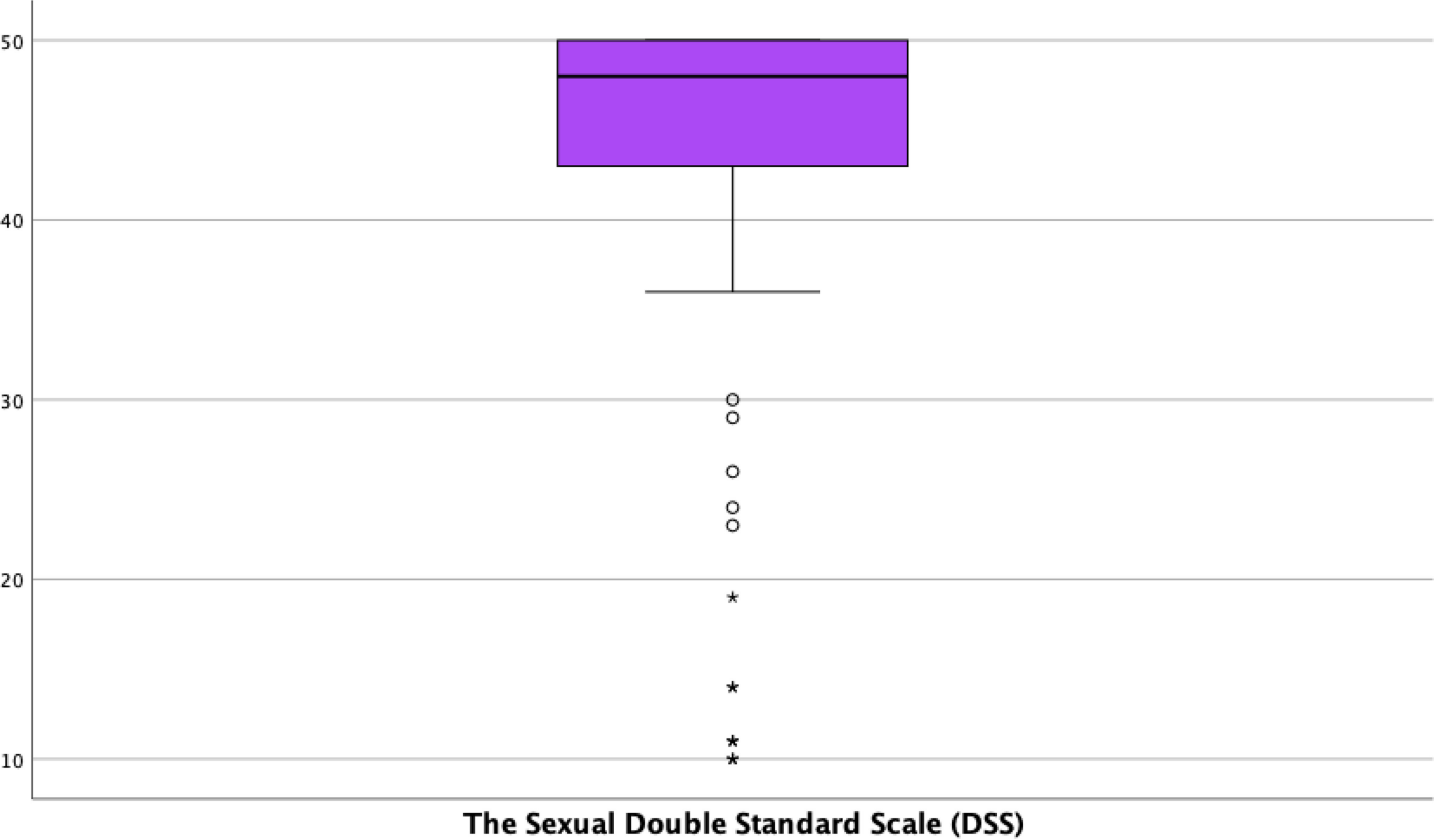
Boxplot of scoring. The Sexual Double Standard Scale (DSS).

Next, when evaluating double standards towards sexuality, a mean DSS score of 30.35 (SD = 9.93) was observed. The distribution of scores by items in the questionnaire can be consulted in Table S3, and the mean distribution of scores can be seen in Figure 3. The mean score in the DSS shows a statistically significant association with alcohol consumption (*p* = 0.001). with means of 45.62 (SD = 8.20) for sporadic consumption, 37.81 (SD = 13.87) for no consumption and 50.00 (SD = 0.0) for frequent consumption. Suffering from some chronic disease (*p* = 0.021) also shows an association with a mean score of 44.29 (SD = 9.50) for the absence of pathology and 36.92 (SD = 16.53) for its presence.

Finally, the relationship between the different scales was studied, observing a statistically significant correlation between the three scales. Specifically, the ATTS was positively correlated with the DSS (Pearson's *r* = 0.487; *p* < 0.001), while the RSAS was negatively correlated with the ATTS (Pearson's *r* = −0.579; *p* < 0.001) and DSS (Pearson's *r* = −0.494, *p* < 0.001).

## 4 DISCUSSION

Nursing students' attitudes towards sexuality, prior to receiving training in sexual and reproductive health, are influenced by their religious beliefs; the higher the religiosity and degree of commitment to religious practice, the lower the degree of liberalism and positive attitudes towards sexuality. Alcohol consumption also shows an association, with sporadic consumption being a predictor of a higher degree of liberalism and attitude towards sexuality. The attitude towards rape, rapists and victims is influenced by whether the student has children, and in this study, desensitization towards the victim and acceptance of the aggressors' behaviour was observed to increased. It can also be seen that the absence of alcohol consumption is associated with the acceptance of rape and its associated behaviours, which also occurs in those students whose family residence is larger than 10,000 inhabitants. That is, the greater the number of inhabitants, the greater the degree of acceptance of sexual assault and its associated behaviours, with the averages descending as the number of inhabitants decreases. Regarding sexual double standards, sporadic and frequent alcohol consumption increases adherence to the sexual double standard, as does the absence of chronic pathologies.

The answers to the questionnaire were collected before receiving sexual and reproductive health training during the nursing degree, thus avoiding the possible influence that this training could have had on the results (O'Connor, 2022; Timmins, 2015). As this study dealt with sensitive data such as sexual identity, declared gender or attitudes towards different behaviours and sexual adherences, it had been decided to anonymize the questionnaire from the collection and to remove elements that would allow the identification of the student; thus, the answers are more honest and he responses that are socially accepted or expected on such a controversial topic minimized. Our sample had a mean age and profile representative of Spain's undergraduate nursing student population (Ministerio de Universidades, 2021). The questionnaires used: ATSS (Ruibal et al., 2005) DSS (Diéguez, 2003; Ruibal et al., 2005) and the RSAS (Sierra et al., 2007) have been validated in a population similar to that of the study.

The mean score on the ATSS was 109.60. Alonso‐Martínez et al. (2022) found a mean score five points higher in their sample of 187 undergraduate nursing students. Although these authors differentiate by year (first year and fourth year), similarities are still present in the scores of both groups. For the study of perceptions of rape, rapists and their victims, an average score on the RSAS of 43.54 was found. Sierra et al. (2007) obtained lower scores than ours, especially for women (30.93) compared to men (40.16). When evaluating double standards towards sexuality, a mean score on the DSS of 30.35 was observed. Alonso‐Martínez et al. (2022) found lower scores reaching a difference of almost half of ours (17.53), meaning they had lower adherence to the greater degree of sexual freedom for men than for women. Other authors, however, find average scores higher than ours by 10 points in the case of university men and more than 13 points in university women (Sierra et al., 2007). Uğurlu and Karahan (2022) found that being female also affects sexual knowledge.

Sporadic alcohol consumption appears to be related to a higher degree of liberalism and attitude towards sexuality, which is in line with other authors (Gil‐García et al., 2013). Besides, Gil‐García et al. (2013) find that this degree of liberalism is taken to the extreme of risk, assuming dangerous sexual practices (absence of contraceptive methods or malpractice thereof). On the other hand, it can be seen that the absence of alcohol consumption is associated with a higher degree of acceptance of the aggressor's behaviours of rape and desensitization towards the victim, which contrasts with other authors (Fuentes‐Pumarola et al., 2021). Fuentes‐Pumarola et al. (2021) found in their cross‐sectional descriptive study an association between sexual violence (rape and sexual assault being one of its forms) and alcohol consumption, either due to not being sufficiently aware due to its influence or because of the acceptance of these practices by ceasing in efforts to express rejection. Regarding sexual double standards, sporadic and frequent alcohol consumption increases adherence to the sexual double standard, which other authors find and associate with gender roles (Duman & Zengin Aydin, 2021).

Religious beliefs are related to the degree of liberalism and positive attitudes towards sexuality. These results are in line with Jadoon et al. (2022) who found religious beliefs were barriers in the treatment of sexuality. Other authors have also found a relationship between the reduction of this liberalism and homophobia, with this being increased in those university nursing students who reported being believers of some religion (Kwak et al., 2019; Rowniak, 2015; Schlub & Martsolf, 1999). This may be because religions (regardless of the type) establish a series of moral precepts that make openness and liberalism difficult in the face of sexuality.

Having children has been associated with greater acceptance of rape, rapists and desensitization of the victims. Desensitization with the victim and acceptance of the behaviour of the aggressors increases if they are parents. No previous research has found an association between these two factors, although some authors find associations between having children and being more conservative in sexual or social aspects (Kerry et al., 2022).

The place of residence, if it is an urban centre with more than 10,000 inhabitants, was associated with a greater acceptance of rape, rapists and desensitization of the victims. That is, the greater the number of inhabitants, the greater the degree of acceptance of sexual assault and its associated behaviours, with the scores descending as the number of inhabitants decreases. Perenc et al. (2022) found results in line with ours in medical university students, although the difference between rural and urban areas was minimal. Other authors have observed that the place of residence and its number of inhabitants is a determining factor for sexual beliefs and attitudes, and it is necessary to take it into account in sexual and reproductive health training (Güdül Öz et al., 2022).

The absence of chronic pathologies increases adherence to the sexual double standard, something that has not been identified in previous studies. Several authors agree that more research is needed between sexuality and chronicity (McInnes, 2003; Verschuren et al., 2010) including in the field of nursing (Steinke, 2013).

The student's age was not identified as being associated with the degree of sexual liberalism; however, other authors such as Benton (2021) observed that the older the student, the greater the degree of sexual liberalism, especially in their progress during the degree courses (Benton, 2021).

Strunk (2017) detected in a study carried out in the United States with 297 participants, that those who were part of the group prior to starting the degree have a lower degree of rejection than those who are close to finishing their Nursing studies. Conversely, Güdül Öz et al. (2022) found differences between each course, but there did not seem to be a progression of tolerance reduction throughout the courses. Our study was carried out in the first courses of the nursing degree, and a clear rejection of behaviours related to rape was found in the majority of students.

The results highlight the importance of understanding the student's profile in order to adapt the training (Horsfall et al., 2012; Lefroy et al., 2015; Wilson et al., 2015). Training in sexual and reproductive health in nursing is a priority for various authors (Akalin & Ozkan, 2021; Lewis & Bor, 1994) who demand more respectful, sensitive and culturally competent clinical care with LGBT groups, especially in the field of sexual care and this can be addressed from university education (Carabez et al., 2015; Keuroghlian et al., 2017; Morris et al., 2019).

Having a map of the students' attitudes in a class makes it possible to adapt the training to the characteristics of that student body (Lefroy et al., 2015). Moreover, adapting teaching based on the feedback received will enhance positive learning experiences and lead to applying a pedagogical model that is relevant for its clinical application (Horsfall et al., 2012; Lefroy et al., 2015; Wilson et al., 2015).

## 5 CONCLUSION

Religious beliefs reduce liberalism and positive attitudes towards sexuality, whereas sporadic alcohol consumption increases them. Having children, not consuming alcohol and living in population centres with more than 10,000 inhabitants increase the desensitization of rape victims and the acceptance of aggressor behaviours. Sporadic alcohol consumption and not suffering from chronic diseases increase adherence to the sexual double standard. Committing to a curriculum in nursing training that teaches sexual and reproductive health with respectful, sensitive and culturally competent content is necessary.

## FUNDING INFORMATION

This research received no external funding.

## CONFLICT OF INTEREST STATEMENT

The authors declare no conflict of interest.

## Supporting information


Data S1.

## Data Availability

Data available at reasonable request.
