# Supplementary material for: Nursing students' attitudes towards sexuality before training in sexual and reproductive health
Source: Nurs Open. 2023 Jul 29;10(10):7038–47. doi: 10.1002/nop2.1959 (PMC10495724; doi:10.1002/nop2.1959)
Supplement: Supplementary file 1 — Data S1. [file NOP2-10-7038-s001.docx]

Supplementary table 1. Attitudes Toward Sexuality Scale (ATSS). Distribution of score by ítems.

| Item | Attitudes Toward Sexuality Scale (ATSS) | | | | |
| --- | --- | --- | --- | --- | --- |
|  | Completely disagree  N (%) | Disagree  N (%) | Neither agree nor disagree  N (%) | Agree  N (%) | Completely agree  N (%) |
| 1. Nude places should be made completely illegal | 90 (76.3) | 15 (12.7) | 9 (7.6) | 3 (2.5) | 1 (0.8) |
| 2. Abortion should be accepted when a woman is convinced that it is her best decision | 4 (3.4) | 6 (5.1) | 14 (11.9) | 25 (21.2) | 69 (58.5) |
| 3. Anyone who is going to engage in coital behavior should be given information and advice on contraception | 1 (0.8) | 1 (0.8) | 0 (0.0) | 18 (15.3) | 98 (83.1) |
| 4. Mothers and fathers of children under 18 who go to a family planning center to obtain contraception should be informed | 7 (5.9) | 11 (9.3) | 32 (27.1) | 15 (12.7) | 53 (44.9) |
| 5. Our government should be tougher on pornography (to prevent its distribution) | 3 (2.5) | 7 (5.9) | 22 (18.6) | 32 (27.1) | 54 (45.8) |
| 6. Prostitution should be legalized | 54 (45.8) | 14 (11.9) | 28 (23.7) | 9 (7.6) | 13 (11.0) |
| 7. Hugging and kissing a person of the other sex is part of the natural growth process of young people | 9 (7.6) | 7 (5.9) | 19 (16.1) | 27 (22.9) | 56 (47.5) |
| 8. Caresses that sexually stimulate any part of the body or all of it are immoral conduct unless it is with the person with whom you are married. | 84 (71.2) | 17 (14.4) | 7 (5.9) | 3 (2.5) | 7 (5.9) |
| 9. Premarital intercourse among young people is unacceptable to me | 97 (82.2) | 7 (5.9) | 5 (4.2) | 3 (2.5) | 6 (5.1) |
| 10. Intercourse between unmarried young people is acceptable to me if there is affection between them and if they both agree | 14 (11.9) | 3 (2.5) | 8 (6.8) | 17 (14.4) | 76 (64.4) |
| 11. Homosexual behavior is an acceptable form of sexual preference | 4 (3.4) | 2 (1.7) | 4 (3.4) | 11 (9.3) | 96 (81.4) |
| 12. A person who gets infected with a sexually transmitted disease gets exactly what he deserves | 78 (66.1) | 23 (19.5) | 12 (10.2) | 3 (2.5) | 2 (1.7) |
| 13. A person's sexual behavior is their business, and no one should make value judgments about it | 1 (0.8) | 4 (3.4) | 9 (7.6) | 20 (16.9) | 84 (71.2) |
| 14. Intercourse should only take place between people who are married to each other | 97 (82.2) | 10 (8.5) | 3 (2.5) | 2 (1.7) | 6 (5.1) |
| 15. Sex education is necessary both in the family and at school | 3 (2.5) | 1 (0.8) | 5 (4.2) | 10 (8.5) | 99 (83.9) |
| 16. If a young person masturbates, they can suffer bad consequences | 91 (77.1) | 10 (8.5) | 8 (6.8) | 3 (2.5) | 6 (5.1) |
| 17. Having sexual fantasies is inadvisable for young people | 89 (75.4) | 15 (12.7) | 5 (4.2) | 3 (2.5) | 6 (5.1) |
| 18. Sex education is dangerous | 100 (84.7) | 8 (6.8) | 1 (0.8) | 1 (0.8) | 8 (6.8) |
| 19. Sexual fantasies are very normal among young people | 1 (0.8) | 4 (3.4) | 13 (11.0) | 30 (25.4) | 70 (59.3) |
| 20. Young people who masturbate carry out a natural and normally healthy behavior | 4 (3.4) | 2 (1.7) | 13 (11.0) | 22 (18.6) | 77 (65.3) |
| 21. Youth should never have access to pornography | 14 (11.9) | 28 (23.7) | 34 (28.8) | 22 (18.6) | 20 (16.9) |
| 22. Young people are, in general, very irresponsible | 37 (31.4) | 30 (15.4) | 36 (30.5) | 9 (7.6) | 6 (5.1) |
| 23. The freedom that young people have today is good | 7 (5.9) | 8 (6.8) | 34 (28.8) | 30 (25.4) | 39 (33.1) |
| 24. In general, I have a good opinion of today's young people | 3 (2.5) | 9 (7.6) | 41 (34.7) | 41 (34.7) | 24 (20.3) |
| 25. It is good that young people can freely access pornographic materials | 35 (29.7) | 29 (24.6) | 39 (33.1) | 8 (6.8) | 6 (5.1) |
| 26. Most young people are responsible | 7 (5.9) | 12 (10.2) | 40 (33.9) | 45 (38.1) | 14 (11.9) |
| 27. Young people today have too much freedom | 22 (18.6) | 28 (23.7) | 39 (33.1) | 19 (16.1) | 10 (8.5) |
| 28. Today's youth deserves a bad opinion | 52 (44.1) | 28 (23.7) | 28 (23.7) | 4 (3.4) | 6 (5.1) |

Supplementary table 2. Rape Supportive Attitude Scale (RSAS). Distribution of score by ítems.

| Item | Rape Supportive Attitude Scale (RSAS) | | | | |
| --- | --- | --- | --- | --- | --- |
|  | Completely disagree  N (%) | Disagree  N (%) | Neither agree or disagree  N (%) | Agree  N (%) | Completely agree  N (%) |
| 1. The fact of being abused is a sexual stimulus for many women | 77 (65.3) | 16 (13.6) | 16 (13.6) | 9 (7.6) | 0 (0.0) |
| 2. A man has some justification for forcing a woman to have sex if she allowed him to believe that she would sleep with him | 106 (89.8) | 4 (3.4) | 4 (3.4) | 3 (2.5) | 1 (0.8) |
| 3. The degree of a woman's opposition should be the most important factor in determining whether a rape has occurred | 27 (22.9) | 21 (17.8) | 15 (12.7) | 21 (17.8) | 34 (28.8) |
| 4. The reason many rapists commit rape is for sex. | 21 (17.8) | 9 (7.6) | 36 (30.5) | 30 (25.4) | 22 (18.6) |
| 5. If a girl starts kissing the neck and caressing, and then he can't control it anymore, it's her fault if her partner forces her to have sex | 95 (80.5) | 12 (10.2) | 10 (8.5) | 1 (0.8) | 0 (0.0) |
| 6. Many women falsely report being raped because they are pregnant and want to protect their reputation | 48 (40.7) | 29 (24.6) | 33 (28.0) | 6 (5.1) | 2 (1.7) |
| 7. It is somehow justified for a man to force a woman to have sex with him if she allowed him to come to her house. | 106 (89.8) | 5 (4.2) | 3 (2.5) | 3 (2.5) | 1 (0.8) |
| 8. Sometimes, the only way a man can arouse a cold (frigid) woman is through the use of force | 107 (90.7) | 6 (5.1) | 4 (3.4) | 1 (0.8) | 0 (0.0) |
| 9. An accusation of rape two days after the event probably isn't really rape | 100 (84.7) | 9 (7.6) | 6 (5.1) | 2 (1.7) | 1 (0.8) |
| 10. A raped woman is a less desirable woman. | 107 (90.7) | 3 (2.5) | 4 (3.4) | 1 (0.8) | 3 (2.5) |
| 11. Somehow, it is justified for a man to force a woman to have sex with him if they have already had sex in the past | 109 (92.4) | 4 (3.4) | 2 (1.7) | 2 (1.7) | 1 (0.8) |
| 12. To protect the man, it should be difficult to prove that a rape has occurred | 79 (66.9) | 17 (14.4) | 17 (14.4) | 3 (2.5) | 2 (1.7) |
| 13. Often, a woman will pretend that she does not want to have sex because she does not want to appear easy, but really she will be waiting for the man to force her. | 97 (82.2) | 11 (9.3) | 7 (5.9) | 3 (2.5) | 0 (0.0) |
| 14. A woman who is conceited and who thinks she is too attractive to talk to men deserves a lesson | 103 (87.3) | 11 (9.3) | 3 (2.5) | 1 (0.8) | 0 (0.0) |
| 15. One reason women falsely report rape is that they often need attention. | 94 (79.7) | 10 (8.5) | 8 (6.8) | 5 (4.2) | 1 (0.8) |
| 16. In most rapes, the victim is promiscuous or has a bad reputation | 99 (83.9) | 11 (9.3) | 5 (4.2) | 2 (1.7) | 1 (0.8) |
| 17. Many women have a hidden desire to be raped and may unconsciously create a situation where there is the possibility of being sexually assaulted. | 106 (89.8) | 7 (5.9) | 3 (2.5) | 2 (1.7) | 0 (0.0) |
| 18. Rape is the expression of an uncontrollable desire for sex | 77 (65.3) | 15 (12.7) | 15 (12.7) | 8 (6.8) | 3 (2.5) |
| 19. It is somehow justified for a man to force a woman to have sex with him if they have been dating for a long time | 109 (92.4) | 3 (2.5) | 4 (3.4) | 1 (0.8) | 1 (0.8) |
| 20. The rape of a woman by a man she knows can be defined as a "woman who later changed her mind" | 103 (87.3) | 9 (7.6) | 4 (3.4) | 1 (0.8) | 1 (0.8) |

Supplementary table 3. The Sexual Double Standard Scale (DSS). Distribution of score by ítems.

| Item | The Sexual Double Standard Scale (DSS) | | | | |
| --- | --- | --- | --- | --- | --- |
|  | Completely agree  N (%) | Agree  N (%) | Neither agree or disagree  N (%) | Disagree  N (%) | Completely disagree  N (%) |
| 1. A woman is expected to be less sexually experienced than her partner | 12 (10.2) | 6 (5.1) | 11 (9.3) | 19 (16.1) | 70 (59.3) |
| 2. A woman who is sexually active is less likely to be desired as a partner | 10 (8.5) | 4 (3.4) | 6 (5.1) | 16 (13.6) | 82 (69.5) |
| 3. A woman should never appear to be ready for a sexual encounter. | 11 (9.3) | 6 (5.1) | 8 (6.8) | 15 (12.7) | 78 (66.1) |
| 4. It is important for men to be sexually experienced in order to teach women | 9 (7.6) | 2 (1.7) | 4 (3.4) | 12 (10.2) | 91 (77.1) |
| 5. A “good” woman would never have a one-night stand, but a man is expected to. | 11 (9.3) | 3 (2.5) | 6 (5.1) | 5 (4.2) | 93 (78.8) |
| 6. It is important for a man to have multiple sexual encounters to gain experience. | 9 (7.6) | 4 (3.4) | 4 (3.4) | 9 (7.6) | 92 (78.0) |
| 7. In sex, the man should take the dominant role and the woman the passive role | 11 (9.3) | 3 (2.5) | 4 (3.4) | 7 (5.9) | 93 (78.8) |
| 8. It is acceptable for a woman to have her condoms | 89 (75.4) | 11 (9.3) | 3 (2.5) | 2 (1.7) | 13 (11.0) |
| 9. It is worse for a woman to be promiscuous than for a man | 11 (9.3) | 3 (2.5) | 10 (8.5) | 9 (7.6) | 85 (72.0) |
| 10. It is the man's decision to start sex | 10 (8.5) | 2 (1.7) | 4 (3.4) | 6 (5.1) | 96 (81.4) |
